# Action verb processing specifically modulates motor behaviour and sensorimotor neuronal oscillations

**DOI:** 10.1038/s41598-019-52426-9

**Published:** 2019-11-05

**Authors:** Anne Klepp, Hanneke van Dijk, Valentina Niccolai, Alfons Schnitzler, Katja Biermann-Ruben

**Affiliations:** 1 Institute of Clinical Neuroscience and Medical Psychology, Medical Faculty, Heinrich Heine University, 40225 Düsseldorf, Germany; 2 Research Institute Brainclinics, 6524 AD Nijmegen, Netherlands

**Keywords:** Language, Motor cortex

## Abstract

Understanding action-related language recruits the brain’s motor system and can interact with motor behaviour. The current study shows MEG oscillatory patterns during verb-motor priming. Hand and foot verbs were followed by hand or foot responses, with faster reaction times for congruent conditions. In ROIs placed in the hand/arm and foot/leg portions of the sensorimotor cortex, this behavioural priming effect was accompanied by modulations in MEG oscillatory patterns preceding the responses. Power suppression in the alpha/beta frequency bands was reduced in congruent conditions in the body-part-specific ROIs. These results imply that the verb-motor priming effect may be a direct consequence of motor cortex contributions to action word processing.

## Introduction

There is accumulating evidence for the flexible recruitment of sensorimotor brain areas during action-related language processing, in line with the idea of embodied and grounded cognition^1^. This is assumed to result from the grounding of conceptual and language processing in sensorimotor experience^2^. Experimentally, reading or hearing action verbs or sentences is accompanied by activation in the motor system as shown by functional magnetic resonance imaging (fMRI)^3,4^. This activation can be somatotopical, arguing for its specificity^3^. Magnetoencephalography (MEG) and Electroencephalography (EEG) with their high temporal resolution find this type of activation early in the processing stream^5–7^ and in neuronal oscillations, namely in the alpha (8–12 Hz) and beta (13–30 Hz) frequencies^8–12^. Moreover, the specificity of motor system recruitment during the processing of action-related language can be inferred from the interaction between language understanding and motor behaviour. For instance, reaction times or response kinematics can be modulated by action verbs or sentences^13–17^. The interaction of action language processing with concurrent motor behaviour is assumed to reflect semantic processing in the brain’s motor system^18,19^. If a motor act is modulated by understanding words or sentences with a specific action content, this may imply that motor execution and language processing partly recruit the same or connected cell assemblies. Thus, identifying the specific effects of language-motor interaction is informative regarding the involvement of sensorimotor areas in conceptual processing^20,21^.

To this end, the current study extends previous research by combining behavioural language-action priming and specific neurophysiological measures. Generally, perceptual repetition priming is associated with both facilitated responses and reduced neuronal activation^22,23^. The neurophysiological mechanism described as repetition suppression, i.e. reduced neuronal responses to repeated stimuli, may also play a role in semantic priming, the processing of stimuli with overlapping semantic features^24,25^. Indeed, reduced hemodynamic responses have been observed for semantically related compared with unrelated verbal stimuli^24–26^, and semantic priming has been linked to a reduction in the N400 component of event-related potentials^27,28^. The processing characteristics of a cortical region can be inferred by investigating its adaptation dynamics in a priming paradigm^26^. In the context of embodied cognition, observing semantic priming effects in sensorimotor areas implies the contribution of neuronal circuits in sensorimotor networks to conceptual and language processing^1,2,29^. For the current experiment, single action verb stimuli describing either hand or foot actions were combined with hand and foot responses. The crucial manipulation was the congruence of the verbs’ implied effector and the response effector. For congruent verb-response conditions, activation of partially overlapping areas in the sensorimotor network was expected to lead to behavioural facilitation and altered neurophysiological responses [^30,31^, for comparison]. In a previous behavioural study with a similar design, we indeed found faster responses following verbs describing actions with the same effector^17^. This paradigm allows the investigation of double dissociation effects within the same subjects within one session, since the response effector is determined by a nonverbal cue, in this case a colour change. Body-part-specific verb-motor priming was also found when responding to verbal stimuli themselves in between-subjects or between-sessions designs^13,21,32,33^ and with overt categorisation of verb effector^15^. Few studies have combined a motor-language interaction paradigm with neurophysiological measurements. One experiment found reduced somatotopic motor activation in event-related potentials (ERPs) for mouth and foot action verbs after priming by a related action sound, compared with an unprimed condition^34^. When a finger or foot response preceded hand and foot verb understanding, reduced activation was found in event-related fields (ERFs) using MEG^29^.

The current experiment extends previous findings by focusing on the association between behavioural priming effects and neurophysiological measures in a previously established paradigm^16,17^. Neuronal oscillations in the alpha and beta band are particularly suitable for investigating this kind of interaction since they are related to motor processing^35,36^. A characteristic pattern of oscillatory power suppression accompanies motor preparation and execution and is thought to reflect neuronal activation, to be followed by a power rebound after movement offset^35,37–39^. Alpha and beta band oscillations have previously been described to also show power modulations during movement observation^38,39^, motor imagery^40,41^, and during the understanding of action-related language^8,10,11,42,43^. Moreover, there is trial-by-trial temporal uncertainty of the transition from verb understanding and categorisation to motor preparation and execution in reaction-time-based paradigms like the current one. While a separate analysis of stimulus-locked and response-locked data may partly overcome this issue^7^, modulations in ongoing neuronal oscillations may be even better suited for detecting effects than strictly time-locked ERP/ERF components. Thus, our design aimed at identifying modulations of the strong sensorimotor rhythms during response preparation and execution by verb processing.

To this end, neuronal oscillations measured by MEG were analysed in regions of interest (ROI) defined in the sensorimotor system and in fronto-temporal language regions. ROI selection was based on previous results in the literature regarding embodied language processing^7,29,44^. While some studies reported bilateral effects of motor resonance in embodied language processing^3^, some found results restricted to the left hemisphere^45,46^. This may reflect left hemispheric language dominance, or possibly the direct motor experience of predominantly right-handed subjects^4^. For the current study, the combination of language processing with overt motor execution rendered the inclusion of only left-hemispheric areas, i.e. contralateral to the movements, feasible. More specifically, sensorimotor ROIs were placed in the upper limb (referred to as “hand”) and lower limb (referred to as “foot”) portions of motor and sensory cortex. Moreover, an ROI was defined in the inferior parietal lobule (IPL). This region is part of the motor cognition network and was previously reported to be sensitive to action-related language processing^7,47^. The inclusion of this ROI was based on the possibility that the verb-motor priming in the current study would affect higher order motor areas such as IPL in addition to primary sensorimotor areas. We also defined ROIs in the classical language areas that might be related to priming, namely in the inferior frontal gyrus (IFG), anterior inferior temporal gyrus (ITG) and posterior superior temporal sulcus (STS)^29^, for a similar approach]. Potential effects in these ROIs could reflect reduced neuronal activation as found in semantic priming using verbal target stimuli^24,25^.

Generally, there is an ongoing debate about the relevance of modality-specific contributions to conceptual processing^48,49^. While some accounts describe how perceptual grounding may form conceptual processing^1,2^, others stress processes of abstraction and the potential for symbolic representation in human cognition^50,51^. With the body of experimental evidence still inconsistent, theoretical discussions have focused on the specific conditions under which embodied aspects of cognition may play a role in a flexible, graded manner^49,52,53^. The current study aims to contribute to the ongoing debate concerning embodied cognition and perceptual grounding by examining novel data of a language-motor interaction experiment. This is not only informative concerning the conditions under which embodied cognition may be observed, and in which manner, but also relevant for future studies in the field. By establishing a link between behavioural and neurophysiological effects of verb-motor priming empirically, further avenues for innovative experimental paradigms that use variations of language-action interaction are opened.

**Figure 1:**
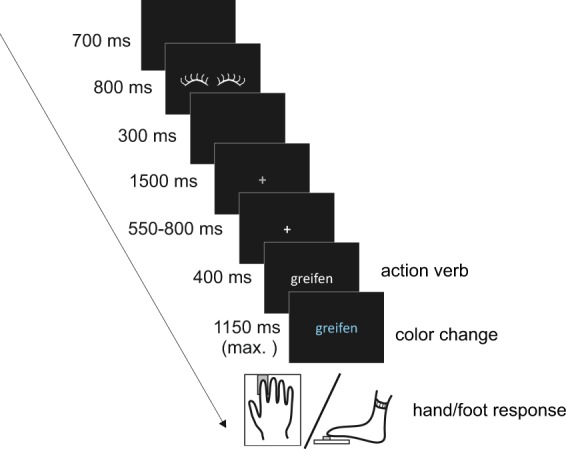
Task design. Following a cue, a single verb was displayed. Verbs could be either a hand, foot, or abstract verb. After 400 ms the font colour changed to either blue or yellow. Only for concrete but not for abstract verbs this colour change had to be followed by a hand or foot response.

In short, the current experiment combined responses with two effectors (hand and foot) and action verbs relating to these effectors in one priming task. Participants read either a hand verb, foot verb, or an abstract verb. A response with either the hand or the foot to a colour change was required only following concrete (i.e., hand and foot) verbs (see Fig. 1). Thus, both hand and foot responses were collected for the same verbal material. Reaction times and neuronal oscillations were investigated regarding differences between primed and unprimed, i.e. congruent and non-congruent verb-response conditions. We expected to find a facilitation of the motor execution in primed conditions, reflected in faster reaction times and reduced oscillatory power suppression.

## Results

**Figure 2:**
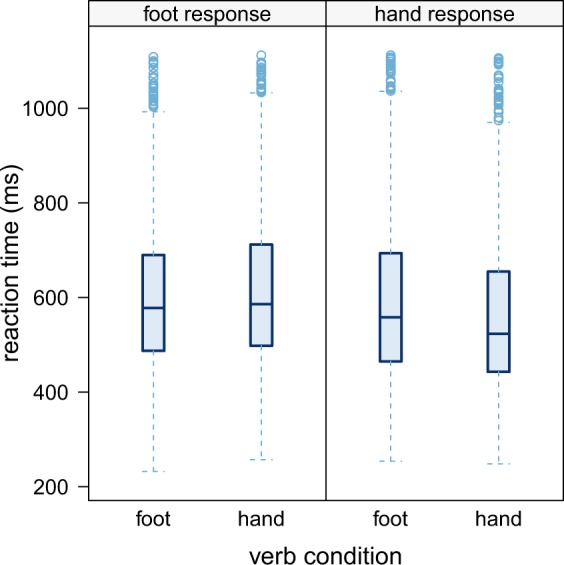
Box-and-whisker-plots of raw reaction time distributions, based on all single trials across participants. Boxes mark 25th and 75th percentile, horizontal bars show median. Whiskers extend to 1.5 interquartile range, dots represent slowest trials. Reaction times were measured from the colour change cue, 400 ms after word onset.

**Figure 3:**
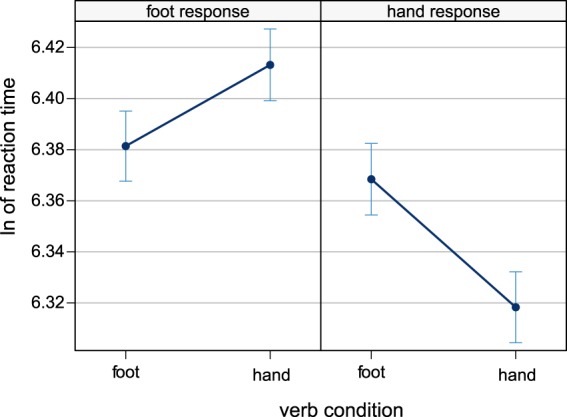
Predictor estimates and confidence intervals for the mixed model on ln-transformed reaction times.

Raw reaction time distributions are shown in Fig. 2 and model estimates in Fig. 3. There was a main effect of response effector, with faster hand responses than foot responses (t = 2.46). This corresponds to mean reaction times of 578 ms (SD = 166) for hand responses and mean reaction times of 611 ms (SD = 160) for foot responses. Moreover, the interaction between verb condition and response effector was also significant (t = −5.18, p < .001). This interaction was followed up by post hoc tests for the effect of verb condition at the two levels of response effector. Hand responses following hand verbs were significantly faster than hand responses following foot verbs (z = 3.075, p < 0.01). This corresponds to mean reaction times of 563 ms (SD = 163) for hand verbs and mean reaction times of 593 ms (SD = 168) for foot verbs. For foot responses, the contrast was also significant with faster responses following foot verbs than hand verbs (z = −2.094, p < 0.04). This corresponds to mean reaction times of 603 ms (SD = 159) for foot verbs and mean reaction times of 618 ms (SD = 161) for hand verbs. Predictor estimates are displayed in Table 1.

**Table 1:** Linear mixed model results for behavioral data. Significant effects are indicated by bold font.

Reaction time					
Predictor	Estimate	Standard Error	df	*t*	*p*
Intercept	6.370	0.036	16.82	147.95	<0.001
verb condition	0.005	0.007	15.90	0.67	0.503
**response effector**	**0.027**	**0.011**	81.44	**2.46**	**0.026**
**verb condition x response effector**	**−0.020**	**0.004**	**16.25**	**−5.18**	<**0.001**
**Accuracy**					
Predictor	Estimate	Standard Error		*z*	*p*
Intercept	3.690	0.167		22.06	<0.001
verb condition	0.059	0.078		0.76	0.882
response effector	0.021	0.141		0.15	0.447
**verb condition x response effector**	**−0.353**	**0.078**		**4.52**	<**0.001**

In the accuracy model, a significant interaction between verb condition and response effector emerged (p < 0.001). This interaction was followed up by post hoc tests for the effect of verb condition at the two levels of response effector. Errors, i.e. responses with in incorrect effector, were significantly less likely to occur when hand responses were required following hand verbs than hand responses following foot verbs (z = −2.762, p < 0.01). This corresponds to a mean overall error rate of 2.50% for hand verbs followed by a hand response prompt and 4.51% for foot verbs followed by a hand response prompt. The reverse contrast was also significant: Errors were significantly less likely to occur when foot responses were required following foot verbs than foot responses following hand verbs (z = 3.596, p < 0.001). This corresponds to a mean overall error rate of 1.95% for foot verbs followed by a foot response prompt and 4.36% for hand verbs followed by a foot response prompt. Predictor estimates are displayed in Table 1. While missed responses and false alarms were not analysed systematically, the mean rates were 2.94% misses/misclassifications (SD = 2.28%) and 10.34% false alarms (SD = 6.51%), indicating a good semantic decision performance with more abstract verbs being classified as concrete than hand or foot verbs being classified as abstract.

**Figure 4:**
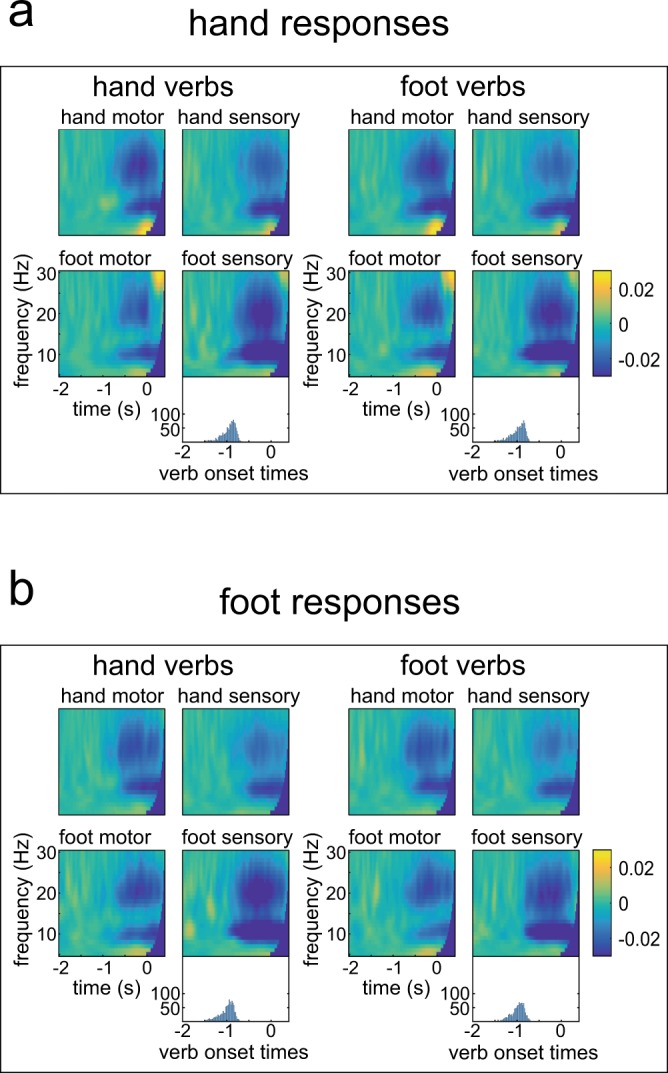
Grandaveraged MEG oscillations in all ROIs for the hand and foot verbs followed by hand or foot responses. Response onset is 0. A histogram of word onset times across all trials and all participants is included for each condition. The response cue had a fixed delay of 400 ms after word onset. Data were baseline-corrected (−2 to −1 s). ITG = inferior temporal gyrus, STS = superior temporal sulcus, IFG = inferior frontal gyrus, IPL = inferior parietal lobule. (**a**) data from trials with hand responses, (**b**) data from trials with foot responses.

Time-frequency data for the hand and foot response conditions and the motor and sensory ROIs are displayed in Fig. 4. Since no significant results were found in ITG, STS, IFG and IPL in any comparison, MEG data from these ROIs are only provided in the Supplementary Information. Descriptively, the characteristic pattern of motor preparatory power suppression in the alpha and beta frequency bands, i.e. power levels below baseline, was observed in sensorimotor cortical areas for both hand and foot responses.

**Figure 5:**
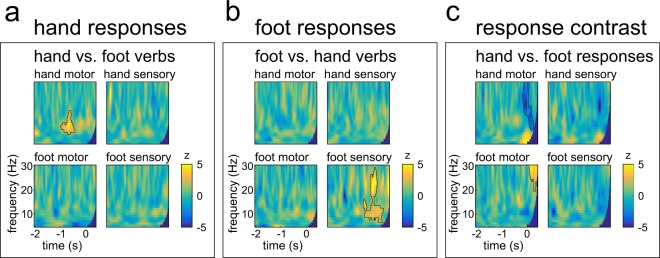
Statistical comparisons of response-locked MEG data. Significant clusters are outlined. Response onset is 0. ITG = inferior temporal gyrus, STS = superior temporal sulcus, IFG = inferior frontal gyrus, IPL = inferior parietal lobule.

For the MEG data, the significant contrasts found in the behavioural results were analysed directly: For hand responses, hand verbs were contrasted with foot verbs; for foot responses, foot verbs were contrasted with hand verbs. Moreover, hand responses were contrasted with foot responses. All results are shown in Fig. 5.

In the statistical comparison of the response contrast, a significant negative cluster was found in the hand motor ROI (p < 0.01, Fig. 5c). Here, alpha and beta power suppression were stronger for hand than for foot responses. The reverse effect was observed in the foot motor ROI with a significant positive cluster (p < 0.05, Fig. 5c) indicating stronger beta power suppression for foot than for hand responses. Both significant clusters emerged around the time of response onset.

The contrast between hand and foot verbs within hand responses yielded a significant positive cluster in the hand motor ROI (p < 0.01). The cluster was significant between about −1000 and −200 ms before response onset and included a frequency range between 9 and 18 Hz (Fig. 5a). Thus, alpha and low beta power suppression was reduced for hand responses preceded by hand verbs, i.e. the congruent condition. In turn, a significant positive cluster for the foot response contrast emerged in the foot sensory ROI (p < 0.01). The significant cluster spread from −800 ms before the response until response execution and included a frequency range between 7 and 27 Hz (Fig. 5b). Analogously to the hand responses, this indicates that alpha and beta power suppression was reduced for the congruent condition. No significant differences emerged in any other ROIs (all p > 0.05). Note also that the analysis time window contained the baseline window, and no significant differences were observed during baseline in any contrast in any ROI.The verb-locked time-frequency data was investigated in a complementary analysis, confirming the timing of significant effects to emerge around response onset in a direct comparison of congruent and incongruent conditions. The details of this analysis are provided in the Supplementary Information. The Supplementary Information also describes data from a whole brain analysis for comparison.

## Discussion

The current study showed a somatotopic verb-motor priming effect in behavioural and neurophysiological measures. Responses were faster and more accurate when they were performed with the same effector as described by a verb. Note that a meaningful assessment of missed responses was impossible since missed responses are indistinguishable from words that were subjectively classified as abstract. Thus, accuracy analysis focused on in/correct responses, not classification errors, and results from the accuracy analysis support the reaction time analysis. With the overall low error rates the conclusions to be drawn from the accuracy analysis are limited, especially since some subjects did not perform any errors in at least one condition. It is noteworthy, however, that no speed-accuracy trade-off seems to have contributed to the pattern of results, since faster conditions also produced less errors. More precisely, hand responses were faster and more accurate following hand verbs than foot verbs, and foot responses were faster and more accurate following foot verbs than hand verbs. This was accompanied by reduced alpha/beta power suppression during the motor preparation stages in the respective portions of the sensorimotor cortex: alpha power suppression was reduced following hand verbs in hand motor cortex, and alpha/beta power suppression was reduced following foot verbs in foot sensory cortex. This reduction of power suppression in sensorimotor oscillations is assumed to reflect reduced neuronal activation, as typically observed for semantic and motor-language priming, where reduced activation is seen as a correlate of facilitated processing^23,29^.

Previous studies using the same verbal material and the same task in a slightly modified design showed similar behavioural effects^16,17^, demonstrating the priming effect to be robust in different subject samples and different experimental settings. The process underlying this facilitation is assumed to be motor resonance of verbal processing as predicted by the embodied cognition hypothesis^1^. Crucially, Niccolai *et al*.^16^ could show that after transcranial direct current stimulation (tDCS) over the hand motor cortex, hand verb processing in this paradigm was influenced. This finding was based on a continuous modulation of motor cortical excitability, which supports the idea that the motor cortex specifically contributes to verb understanding. However, that study was not aimed to investigate the specificity of priming effects in the brain. The current experiment, using MEG, allowed the investigation of the neurophysiological effects within the verb-motor priming paradigm. Indeed, significant differences between congruent and non-congruent conditions emerged in body-part-specific ROIs. In this respect, the current study provides novel insights into language-motor interaction by showing a direct link between behavioural effect and the neuronal oscillatory signatures of motor processing. While the current data cannot establish a causal link between language and motor processing, it adds to the debate of the relevance of sensory-motor contributions to conceptual processing. Not only does the sensorimotor system appear to play a role in verb-motor priming, but it also modulates alpha and beta oscillations, providing information about the brain region as well as a functional mechanism involved in this kind of priming. This supports the idea of a flexible semantic system in interacting modality-specific processing areas, where the processing of action-related language is assumed to include sensorimotor activations under specific circumstances^1,20,53^. In the case of the current study, the semantic processing of single verbs appears to have recruited sensorimotor brain areas strongly enough to result in detectable modulations of the oscillatory motor rhythms as well as affect motor output, i.e. response behaviour. These results also imply that sensorimotor contributions are subtle and that other brain networks are involved in language processing. Nevertheless, it is unclear how the current results could have been observed if action-related language processing was completely amodal.

Although the latency and frequency range of a significant cluster in the context of the permutation based statistics should not be directly interpreted as reflecting a sharply circumscribed effect^54^, the clusters related to the priming effect in the hand compared with the foot response contrast may imply a subtly different underlying effect. The cluster in the hand response contrast emerges earlier, around 1000 ms before the response, compared with around 700 ms before the response in the foot response contrast. The cluster in the hand response contrast also does not last as long as the cluster in the foot response contrast. We did not hypothesise a latency difference between these contrasts. While hand responses are overall faster than foot responses, the mean reaction time difference is only about 30 ms. Moreover, MEG oscillations were analysed with respect to response onset, thus aligning the time scale across conditions. More importantly, the contrasts were performed within, not across response effectors. Different response devices used for the hand and foot responses and longer neuronal conduction times for the lower limbs^55^ may have contributed to slower reaction times for foot responses. However, it is unlikely that this would also lead to a different latency of the observed oscillatory effect with regard to the recorded response triggers. There may nonetheless be a difference in the neuronal correlates of motor preparation for hand and foot verbs in this study. Previous research could show that oscillatory power suppression, or event-related desynchronisation, is observed for different effectors and different types of responses^56^. It is not straightforward to directly quantify these oscillations depending on kinematic parameters such as response velocity, amplitude, force, or the number of muscles involved. More importantly, it may be oversimplifying to assume that the types of responses used in this study should follow the exact same motor preparation. The well-learned lifting of the right index finger may involve neuronal networks preparing a downstream motor command differently than the downward press with the right foot. This could imply that the interface of language processing may target different aspects of motor preparation depending on the type of response and leading to different timecourses regarding the relative differences in the congruence contrast. Another explanation could be that the contributions of sensorimotor hand and foot areas to language processing differ in general. The hand area, showing differences between congruent (i.e. primed) and incongruent (i.e. unprimed) conditions as early as 1000 ms before response onset, might therefore have an earlier contribution to effector specific language processing, maybe in terms of earlier simulation processes than the foot area^5^. Hand sensorimotor areas and hand actions may overall be involved more substantially in language processing than sensorimotor areas representing other body parts, since performing and understanding hand actions is strongly linked to language processing by co-speech gestures^57^. Hand actions could also have contributed to the phylogenetic evolution of communication from gestures to language^58^. Thus, there appear to be differences between language processing in hand and foot sensorimotor areas that are not yet fully understood. Nevertheless, the current study found behavioural and neurophysiological effects of verb-motor priming that are body-part specific, both for hand and foot verbs and brain areas.

Clusters also differed with respect to frequency range. For the foot response contrast, the significant cluster included frequencies in the alpha and beta range from about 7 to 27 Hz. For the hand response contrast, only frequencies between 9 and 18 Hz formed the significant cluster. It is unclear whether this might be due to sampling issues or to low signal-to-noise ratio in a subtle effect, i.e., the current analysis of the current participant sample may have been unable to detect differences in higher frequencies for the hand response contrast. Alternatively, this may reflect a true difference in processing within the hand and foot sensorimotor areas. Even though movement-related desynchronisation and synchronisation patterns are found for different effectors, each primary sensorimotor area seems to have its own intrinsic rhythm^56^ and frequency differences between oscillations related to hand and foot movements have been reported before. For instance, post-movement beta rebound was associated with higher peak frequencies for foot than for hand movements^59^.

Additionally, the priming effects for hand responses were observed in the motor ROI in the precentral gyrus. For foot responses, the priming effect was observed in the ROI labelled as a sensory area in the paracentral lobule. While the brainnetome atlas provides a very fine-grained parcellation of the whole brain^60^, both the use of an atlas-based rather than individually and/or functionally defined ROI selection and the overall limited spatial resolution of MEG may contribute to this discrepancy. Thus, our results should not be interpreted as a claim that verb-motor priming would be associated with motor cortex effects for hand responses but sensory cortex effects for foot responses. A more parsimonious explanation is that our methods could distinguish between two separate effects in the sensorimotor system for hand and foot responses, but that the spatial specificity of these effects in adjacent brain areas should not be overestimated.

When directly contrasting hand and foot responses, hand responses were associated with stronger alpha/beta power suppression in hand motor areas, and foot responses were associated with stronger beta suppression in foot motor areas. These effects are strongest around the time of response onset and shortly after, during the movement-related desynchronisation phase. Thus, the current study was able to detect opposed, specific effects for the two response types, which is an important basis for the interpretation of the priming contrast results.

Apart from the effects in hand and foot sensorimotor cortical areas, the current experiment found no other areas in the language system to be differentially modulated by the congruence contrast in verb-motor priming. Previous studies reported contributions to action language processing and also to priming for instance in the posterior superior temporal lobe^29^ or inferior parietal lobule^7^. However, different experimental paradigms may explain the different results. In the study by Dalla Volta *et al*.^7^, only hand responses were required, while the current study used a choice reaction task with two different effectors. This may have different implications for the involvement of motor network nodes such as the inferior parietal lobule in movement preparation depending on a priming condition. In Mollo *et al*.^29^, motor execution preceded language processing, and the priming effect in the superior temporal lobe was found in relation to verb processing. Similarly, studies investigating semantic or repetition priming found reduced neuronal activation^61,62^ or reduced alpha/beta suppression^63,64^ in temporal areas in response to a related or identical target word following a prime word. This is essentially different from the current study where alpha/beta oscillations were investigated during motor preparation, not language processing alone. Here, effects were found in sensorimotor but not in fronto-temporal language areas. Thus, it is conceivable that priming affecting language processing may primarily be observed in core areas of language processing, while our study found priming effects on motor execution in the motor system. It remains to be determined to what extent areas outside the sensorimotor cortex contribute to verb-motor priming.

Future studies may investigate how adjustments to the experimental design and the task influence the behavioural and neurophysiological effects. For instance, stimulus timing may be a key factor^30,65,66^, as seem task instructions^17^ and the actual combination of language and motor tasks^7,29^. Moreover, using a verb-motor priming paradigm in a sample of patients with movement disorders such as Parkinson’s Disease would be informative regarding the relative contributions of motor areas for language processing^67,68^.

Taken together, the current experiment extends previous findings of the neurophysiological effects of language-motor interaction by investigating effects in the motor domain and with a response selection task. The current results show that language-motor interaction can be body-part specific and associated with modulations in neuronal oscillations. The sensorimotor rhythms in the alpha and beta band during motor preparation reflect a somatotopic priming effect: Congruent verb-response conditions, which are also associated with faster reaction times, show a reduction in alpha/beta suppression during motor preparation only in the sensorimotor cortical area associated with the respective limb. Thus, the common principle of priming as reduced neuronal activation for associated stimuli appears to present as reduced motor preparatory power suppression in this paradigm, where action verbs were able to prime motor responses. These results imply that the verb-motor priming effect may be a direct consequence of motor cortex contributions to action word processing, in line with embodied cognition theories.

## Methods

### Participants

Participants gave written informed consent prior to the experiment and received financial reimbursement. The study is in line with the Declaration of Helsinki and was approved by the ethics committee of the Medical Faculty at Heinrich-Heine-University, Düsseldorf (study number 3400). All subjects were native monolingual speakers of German, right-handed and right-footed, with normal or corrected-to-normal vision, no history of neurological/psychiatric disorders and were not using medication affecting the central nervous system. Right-handedness was confirmed using the German translation of the Edinburgh Handedness questionnaire^69^ and the Hand Dominance Test^70^. The Lateral Preference Inventory^71^ was used to confirm right-footedness. MEG was measured continuously during the experiment. Twenty healthy subjects participated. Three subjects were excluded from the final data analysis: for one subject, MEG data was lost due to a technical failure. The other two subjects did not complete the required structural MRI measurement. All analyses were performed for the final set of 17 subjects (10 female, mean age = 24.88 years, SD = 5.86).

### Stimulus material

The stimulus set was comprised of 48 German hand action verbs, e.g. *greifen (to grasp)*, 48 foot action verbs, e.g. *gehen (to walk)*, and 48 abstract verbs, e.g. *raten (to guess)*. For details on verb selection, rating and matching procedures, please see Klepp *et al*.^6^. Ratings (n = 30, different subjects as this study) had been performed for familiarity and imageability. Crucially, group means for hand and foot verbs did not differ statistically for these variables nor word frequency or length.

### Procedure

Neuromagnetic brain activity was recorded continuously by a 306 channel Neuromag MEG system (Elekta Neuromag, Helsinki, Finland) located at University Hospital Düsseldorf. A sampling rate of 1000 Hz with an online bandpass filter of 0.03–330 Hz was used. Analyses were performed offline. Bipolar skin electrodes were used to record electrooculogram (EOG) and electrocardiac activity for later artefact rejection. Four head position indicator (HPI) coils were fixed to the scalp and their position was digitised (Polhemus Isotrak, Colchester, Vermont, USA) for later coregistration of MEG data with anatomical MRIs.

Participants were seated comfortably in the magnetically shielded room. The experimental procedure was adapted for MEG from Klepp *et al*.^17^ and is shown in Fig. 1. Presentation 14.9 software (Neurobehavioral Systems, Albany, California, USA) was used for stimulus presentation. Verbs were projected in white letters onto a black background for 400 ms. Then the colour of the letters changed to either yellow or blue. This colour change determined the type of required response: One colour was associated with hand responses, the other colour with foot responses. This assignment was counterbalanced across participants. Crucially, participants were instructed to only respond following concrete verbs, i.e. hand and foot verbs, and to refrain from responding for abstract verbs. Hand responses were given using the right index finger on a finger-tapping pad. Foot responses were delivered with the toes of the right foot on a response box. Reaction times were measured from the colour change. After a response or after 1150 ms in case no response occurred, a black screen lasting 700 ms was followed by a pictogram of closed eyes for 800 ms, to indicate an eye blink period, and a black screen for 300 ms. Then, a grey fixation cross was shown for 1500 ms. A white fixation cross, displayed for 550–800 ms, indicated the beginning of a new trial. Each verb-colour combination was shown twice in a pseudorandomised order. A practice run (24 trials) was used, and the 576 trials of the main experiment were split into eight blocks lasting about four minutes and separated by short breaks. One to two weeks after the MEG session, anatomical MRI images were acquired with a 3 T magnetom machine (Siemens, Erlangen, Germany). MRIs were aligned with the MEG coordinate system offline using the HPI coils and anatomical landmarks.

### Data processing

MEG data from 204 planar gradiometers were analysed using Fieldtrip^72^, an open source toolbox for Matlab (Mathworks, Natick, MA, USA). For each subject, faulty MEG channels were excluded. Only correct trials were analysed. Trials were defined from two seconds before verb onset to 0.5 seconds after response onset, resulting in trials of different lengths depending on reaction time. Data epochs contaminated by sensor jumps or muscle artefacts were identified by a semiautomatic artefact detection routine and removed from the trial data. If artefacts occurred between verb and response onset, the whole trial was removed. Line noise was filtered using bandstop filters with a width of 2 Hz centred at 50, 100 and 150 Hz. A lowpass filter at 260 Hz and a highpass filter at 2 Hz were used. Data padding of 10 s around each trial was used for preprocessing and filtering. Independent component analysis was applied and identification of components representing cardiac and eye movement/blink artefacts was supported by a mutual information approach using the peripheral reference electrodes^73^. Artefact components were removed.

The following ROIs were defined using the brainnetome atlas^60^: *hand motor* (with the atlas label “PrG, Left Precentral Gyrus A4ul, area 4 (upper limb region”), *hand sensory* (label “PoG, Left Postcentral Gyrus A1/2/3ulhf, area 1/2/3 (upper limb, head and face region)”), *foot motor* (label “PCL, Left Paracentral Lobule A4ll, area 4, (lower limb region)”), *foot sensory* (label “PCL, Left Paracentral Lobule A1/2/3ll, area 1/2/3 (lower limb region)”), *ITG* (label “ITG, Left Inferior Temporal Gyrus A20r, rostral area 20”), *STS* (label “pSTS, Left posterior Superior Temporal Sulcus cpSTS, caudoposterior superior temporal sulcus”), *IFG* (label “IFG, Left Inferior Frontal Gyrus A45c, caudal area 45”) and *IPL* (label “IPL, Left Inferior Parietal Lobule A40rv, rostroventral area 40 (PFop)”).

**Figure 6:**
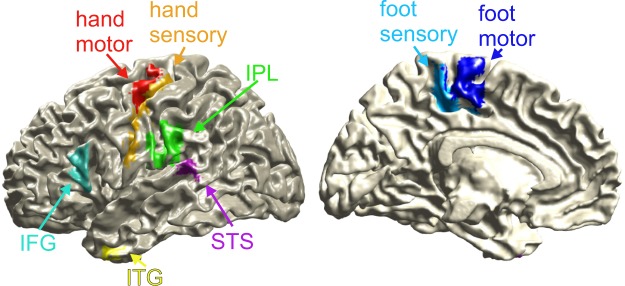
Cortical surface projection of the ROIs. ITG = inferior temporal gyrus, STS = superior temporal sulcus, IFG = inferior frontal gyrus, IPL = inferior parietal lobule. Note that ROIs were defined using labelled 3-dimensional grids and the visualisation is dependent on surface projection parameters.

Individual anatomical MRIs were coregistered with MEG data and the brain volume was discretised to a three-dimensional grid with 1 cm resolution. Gridpoints were warped to the normalised MNI brain, so that for each subject the mapping between brain anatomy parcellated by the brainnetome atlas and gridpoint locations was determined. Linearly constrained minimum variance (LCMV) beamforming was used for source reconstruction of the averaged individual MEG data. The source reconstruction filters derived from this step were used on trial level data and one timecourse per trial for each ROI was obtained by averaging gridpoints in each ROI. These averaged gridpoints were used as virtual sensors for further analyses. To reduce spatial leakage between adjacent sources, an orthogonalisation algorithm was used^74^. A projection of all ROIs onto a standardised brain can be found in Fig. 6.

For the MEG analysis, time-frequency analysis was performed for −2 to 0.5 seconds around response onset. Time-frequency representations (TFRs) for frequencies between 2 and 34 Hz with steps of 2 Hz were computed using a discrete Fourier transformation with a single Hanning taper. This transformation was applied on an adaptive sliding time window with a width of 5 cycles of the respective frequency moving in steps of 25 ms. The time window for trial-wise baseline correction was −2 to −1 s.

### Statistical analysis

For behavioural data analysis, reaction times of correct trials were ln-transformed. Linear mixed-effect models were fit using the *lme4* package^75^ for *R*^76^, including crossed random effects for participants and items. The 2-level factor verb condition and the 2-level factor response effector were modelled as fixed effects. The maximal random effects structure justified by the design was used. Thus, random effects for participants included random intercepts as well as random slopes for the two factors and their interaction. Random intercepts were used for items. Accuracy was assessed by comparing the proportion of correct responses and responses with the incorrect body part. A generalised linear mixed-effect model was fit where verb condition and response effector were modelled as fixed effects. Random intercepts for subjects and items were used as well as random slopes for the interaction of response effector and subject. Factors were sum coded. P-values were estimated using the *afex* package^77^, with the Kendal-Roger method for reaction time data and by parametric bootstrapping for the accuracy data. Post hoc analyses were performed using the *emmeans* package^78^. Here, z-ratios assessed the differences of marginal means of a factor of interest at the levels of another factor. Model results were plotted using the *effects* package^79^.

Statistical analysis of MEG data used a cluster-based randomisation approach^54^ for the contrasts derived from the behavioural analysis. In a first step, single-subject trial-level data was used to obtain t-values for each time-frequency pair in each ROI. These t-values were converted to z-values due to non-equal trial numbers between conditions^80^. Group-level statistics were performed by comparing these z-values against a matrix of zeroes, randomly permuting the two distributions with 1000 repetitions. The resulting time-frequency clusters with cluster-level p-values below 0.05 were considered significant. All ROIs entered the analysis simultaneously, thus correcting for multiple comparisons across virtual channels, but clusters were not restricted to include more than one ROI. Statistical analyses were performed on frequencies between 5 and 30 Hz, for the time window of −2 to 0.3 s around the response. Frequency and time were not averaged. The congruent and incongruent conditions for each response type were contrasted, as well as overall hand and foot responses.

## Supplementary information

Supplementary Information

## Data Availability

The datasets generated during and/or analysed during the current study are available from the corresponding author on reasonable request.
