# Supplementary material for: Action verb processing specifically modulates motor behaviour and sensorimotor neuronal oscillations
Source: Sci Rep. 2019 Nov 5;9:15985. doi: 10.1038/s41598-019-52426-9 (PMC6831701; doi:10.1038/s41598-019-52426-9)
Supplement: Supplementary file 1 — Supplementary Information [file 41598_2019_52426_MOESM1_ESM.pdf]

Supplementary Information for: Action verb processing specifically modulates motor behaviour and sensorimotor neuronal oscillations

Anne Klepp[1], Hanneke van Dijk[2], Valentina Niccolai[1], Alfons Schnitzler[1], Katja Biermann-Ruben[1]

[1]Institute of Clinical Neuroscience and Medical Psychology, Medical Faculty, Heinrich Heine University, 40225 Düsseldorf, Germany
[2]Research Institute Brainclinics, 6524 AD Nijmegen, Netherlands

Results from further ROIs

For the ROIs in ITG, STS, IFG and IPL, descriptive time-frequency data are shown in Fig. S1. The statistical comparisons are shown in Fig. S2, for hand verbs vs. foot verbs within hand response (Fig. S2a), foot verbs vs. hand verbs within foot responses (Fig. 2b) and hand vs. foot responses (Fig. S2c). No significant differences emerged.

Visualization of whole-brain data

While the main analysis focused on specific ROIs, a comprehensive overview of time-freuqency data is presented here. Fig. S3 shows the temporal evolution across the whole sensor space in 100 ms steps. Data processing included the same parameters as the main analysis, i.e. time-frequency analysis for -2 to 0.5 seconds around response onset, for frequencies between 2 and 34 Hz with steps of 2 Hz with a single Hanning taper. The sliding time window had a width of 5 cycles of the respective frequency and moved in steps of 25 ms. The time window for trial-wise baseline correction was -2 to -1 s. Suppl. Fig. 4 shows surface projections centered on the time-frequency window of the significant effects in the main analysis. Here, cortical sources of the grandaveraged relative power differences were estimated using dynamic imaging of coherent sources (Gross, J., Kujala, J., Hamalainen, M., Timmermann, L., Schnitzler, A., Salmelin, R., 2001. Dynamic imaging of coherent sources: studying neural interactions in the human brain. PNAS 98, 694–699). Common spatial filters for each grid point (1 cm resolution) were calculated data from the conditions of interest in the respective time windows and frequencies of interest. The leadfield was based on a realistically shaped singleshell volume conduction model (Nolte, G., 2003. The magnetic lead field theorem in the quasi-static approximation and its use for magnetoencephalography forward calculation in realistic volume conductors. Phys Med Biol 48, 3637–3652.) for individual MRIs. The spatial filters were used to calculate subject-specific relative power differences between conditions, normalised to MNI space. These were grandaveraged and visualised as surface projections (Fig. S4).

Verb-locked analysis

The same parameters as in the response-locked analysis were also used for the analysis of verb-locked data, where time-frequency analysis was performed for -1 to 1.3 seconds around verb onset. The baseline time window was set to -1 to -0.5 s. Baseline correction was performed for the time window of -1 to 1.2 s around the word onset for verb-locked data. For the statistical comparison, the contrasts between hand and abstract verbs, between foot and abstract verbs, between hand and foot verbs, and between congruent and incongruent conditions for each response types were assessed. Additional exploratory analyses were used to further assess the response-locked results in the complementary verb-locked data. To this end, congruent and incongruent conditions were compared in a more restricted time window of 0 to 1.3 s only for the hand motor and foot sensory ROI. The descriptive time-frequency data for the three verb conditions and all ROIs are displayed in Fig. S5. The characteristic power suppression in the alpha and beta frequency bands can be seen in sensorimotor cortical areas, STS and IPL for both hand and foot verbs, which were followed by responses with either the hand or the foot. For the abstract verbs, beta and especially alpha suppression was also observed, but appears weaker than for the hand and foot verbs. In the statistical analysis of verb-locked data, no significant differences emerged between hand and foot verbs in any ROI (all $p > .05$). For the contrasts between hand and abstract verbs and between foot and abstract verbs, a broadband effect emerged around 500 ms after word onset in the motor and sensory ROIs, in STS and in IPL (all $p < .05$, Fig. S6a and S6b). Stronger alpha- and beta-band suppression was observed for hand and foot verbs than for abstract verbs. Despite not reaching significance in the full comparison including all ROIs, the exploratory analysis of the hand motor and foot sensory ROI revealed an analogous pattern to the response-locked analysis: power suppression was lower in congruent than in incongruent conditions, with the significant clusters emerging around the onset of the response cue (Fig. S6c). The significant

differences between hand/foot and abstract verbs that were observed as stronger broadband alpha/beta suppression present as clusters emerging around 500 ms after verb onset and lasting until the end of the analysis window. However, this is not an indication of differences in verbal processing but can be attributed to the fact that responses were only executed following hand and foot verbs. Thus, the significant clusters reflect the contrast between responses and no responses. As already suggested by visually inspecting the time-frequency representations, all motor and sensory ROIs as well as STS and IPL captured the response-associated oscillatory power suppression strongly enough to be statistically significant, with hand motor and foot motor ROIs showing the most prominent clusters.

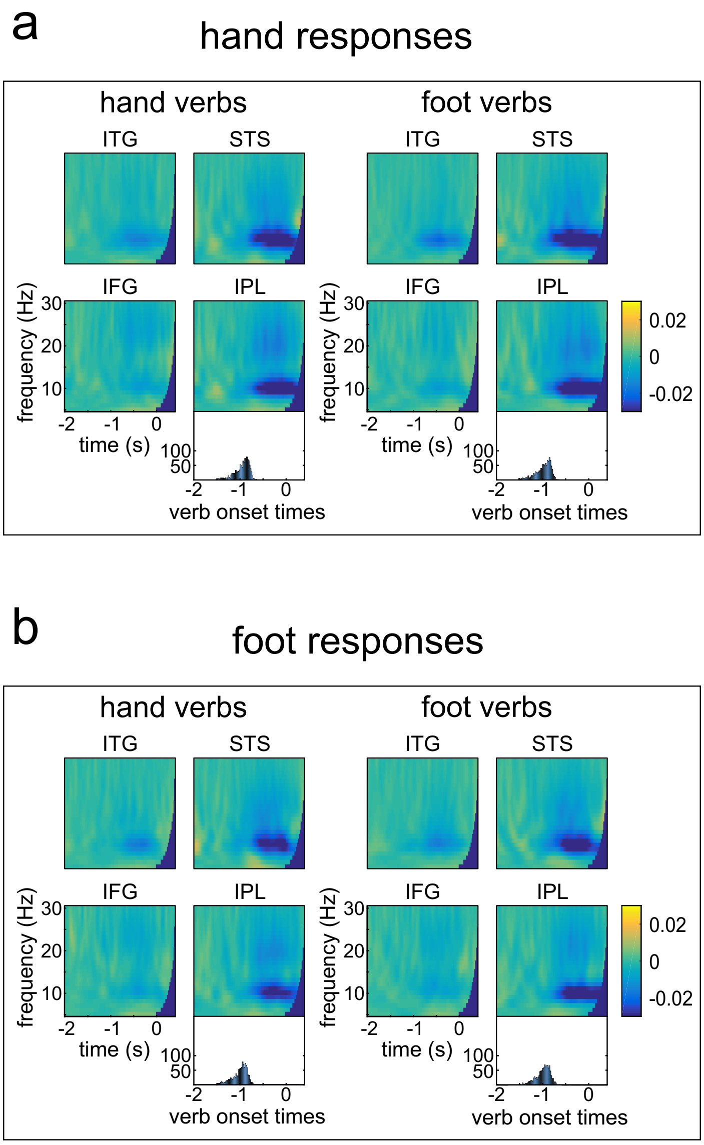

Figure S1: Grandaveraged MEG oscillations in ITG, STS, IFG and IPL for the hand and foot verbs followed by hand or foot responses. Response onset is 0. A histogram of word onset times across all trials and all participants is included for each condition. The response cue had a fixed delay of 400 ms after word onset. Data were baseline-corrected (-2 to -1s). ITG = inferior temporal gyrus, STS = superior temporal sulcus, IFG = inferior frontal gyrus, IPL = inferior parietal lobule.

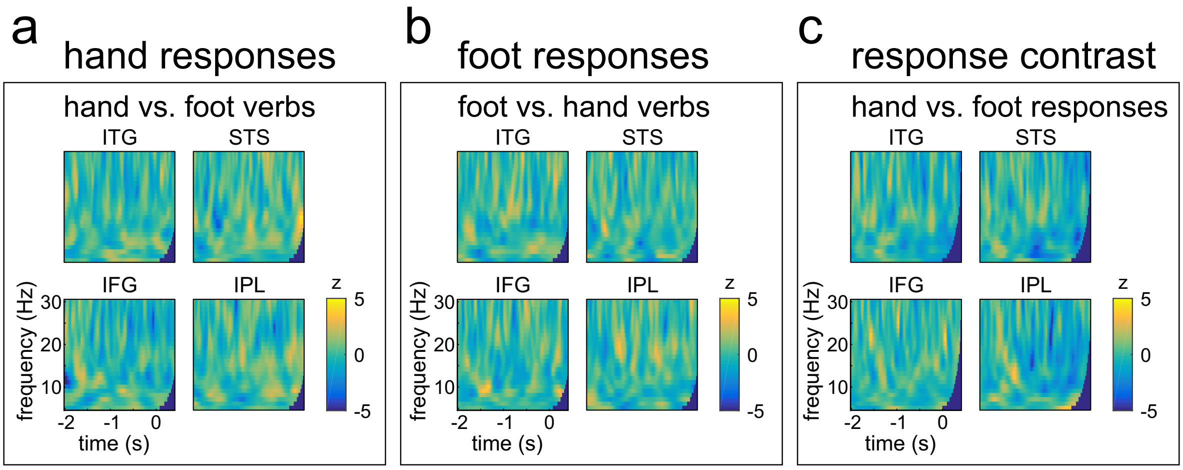

Figure S2: Statistical comparisons of response-locked MEG data. There were no significant clusters. Response onset is 0. ITG = inferior temporal gyrus, STS = superior temporal sulcus, IFG = inferior frontal gyrus, IPL = inferior parietal lobule.

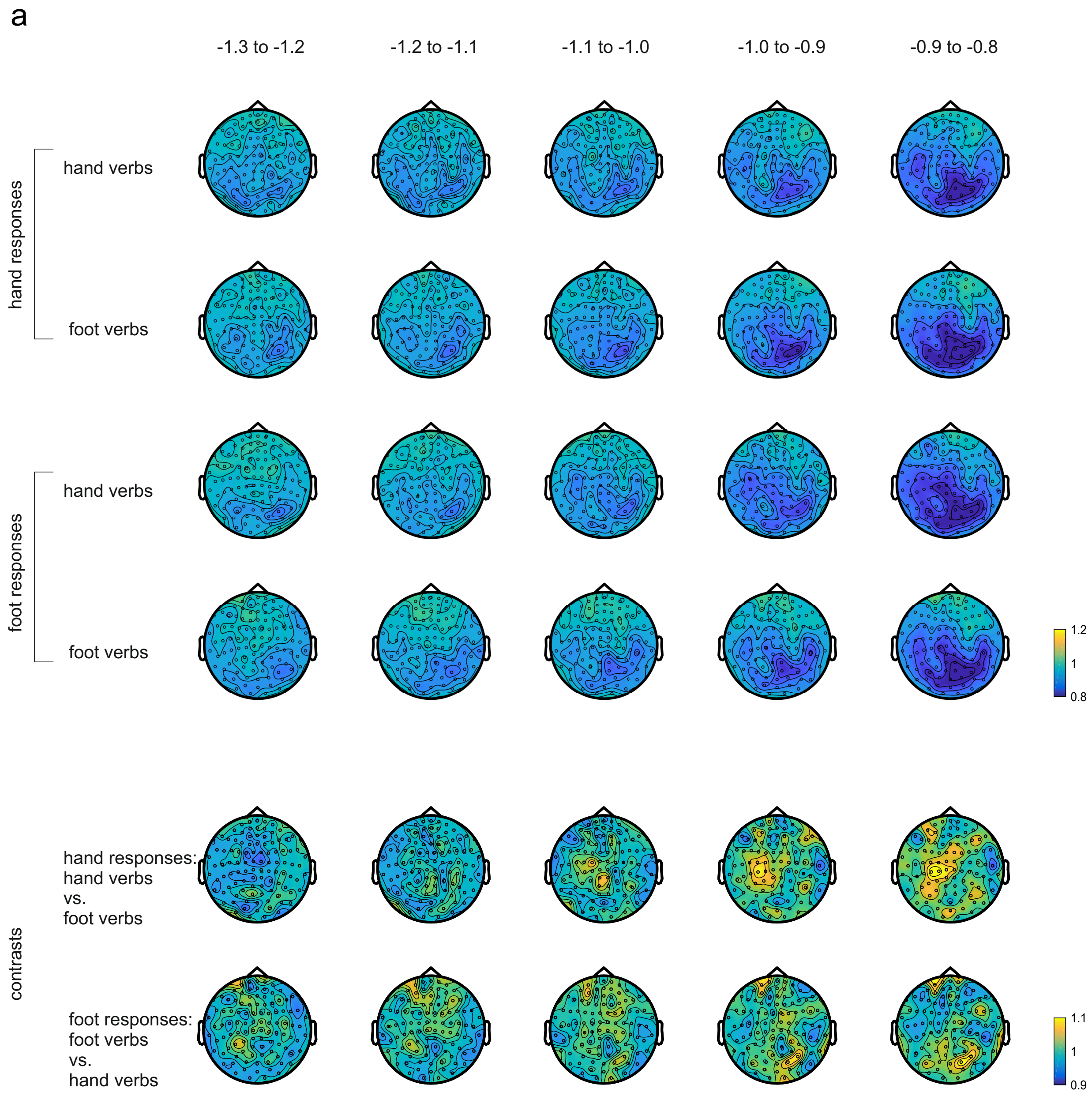

Figure S3: Grandaveraged time-frequency data in all MEG sensors, from -1.3 to 0.2 s in steps of 100 ms, in all four verb-response conditions as well as the contrasts of hand vs. foot verbs within hand responses and foot vs. hand verbs in foot responses. Time is given in seconds before response onset. a-c: alpha band (7-13 Hz), d-f: beta band (20-30 Hz).

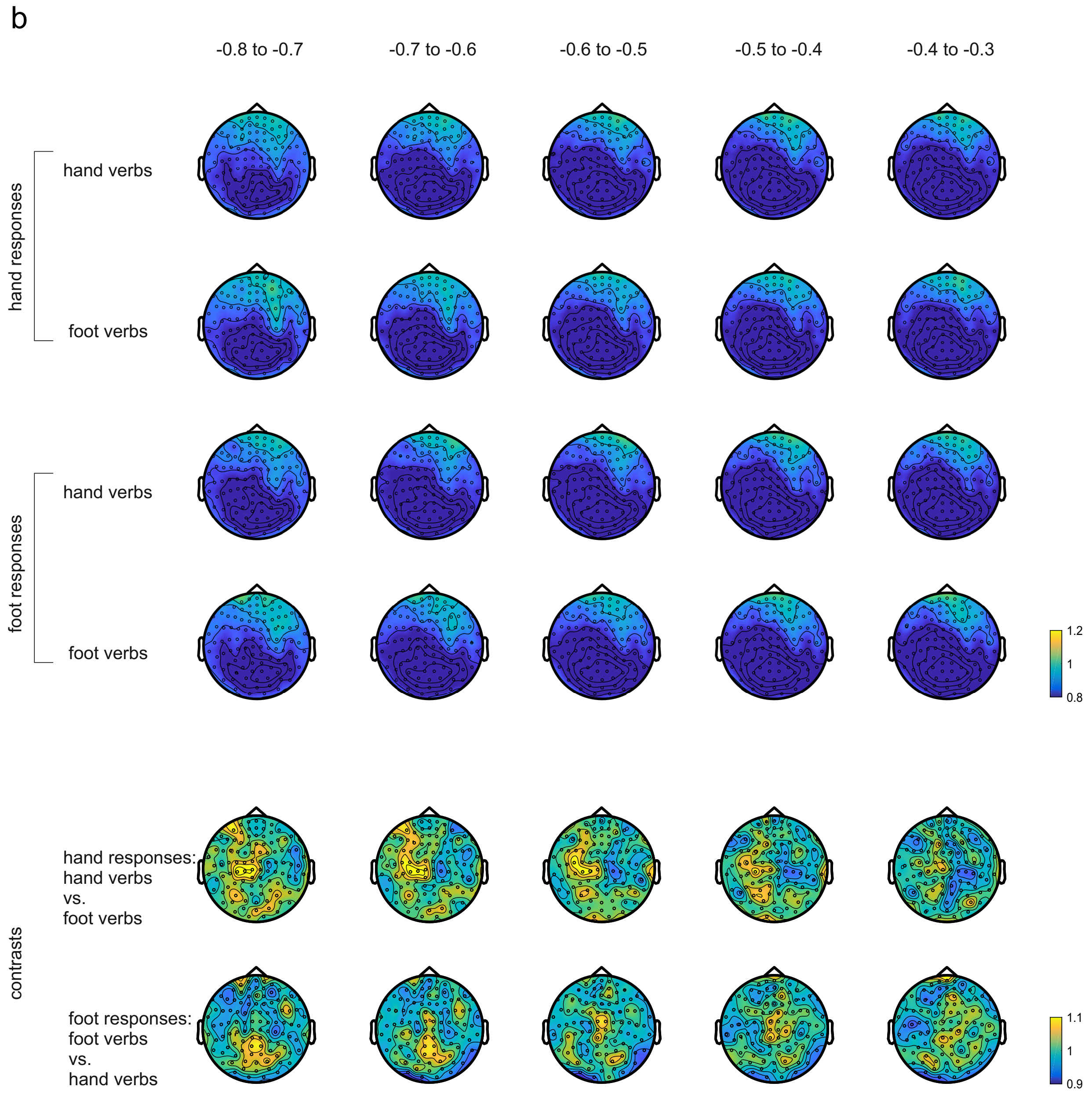

Figure S3: Grandaveraged time-frequency data in all MEG sensors, from -1.3 to 0.2 s in steps of 100 ms, in all four verb-response conditions as well as the contrasts of hand vs. foot verbs within hand responses and foot vs. hand verbs in foot responses. Time is given in seconds before response onset. a-c: alpha band (7-13 Hz), d-f: beta band (20-30 Hz).

c

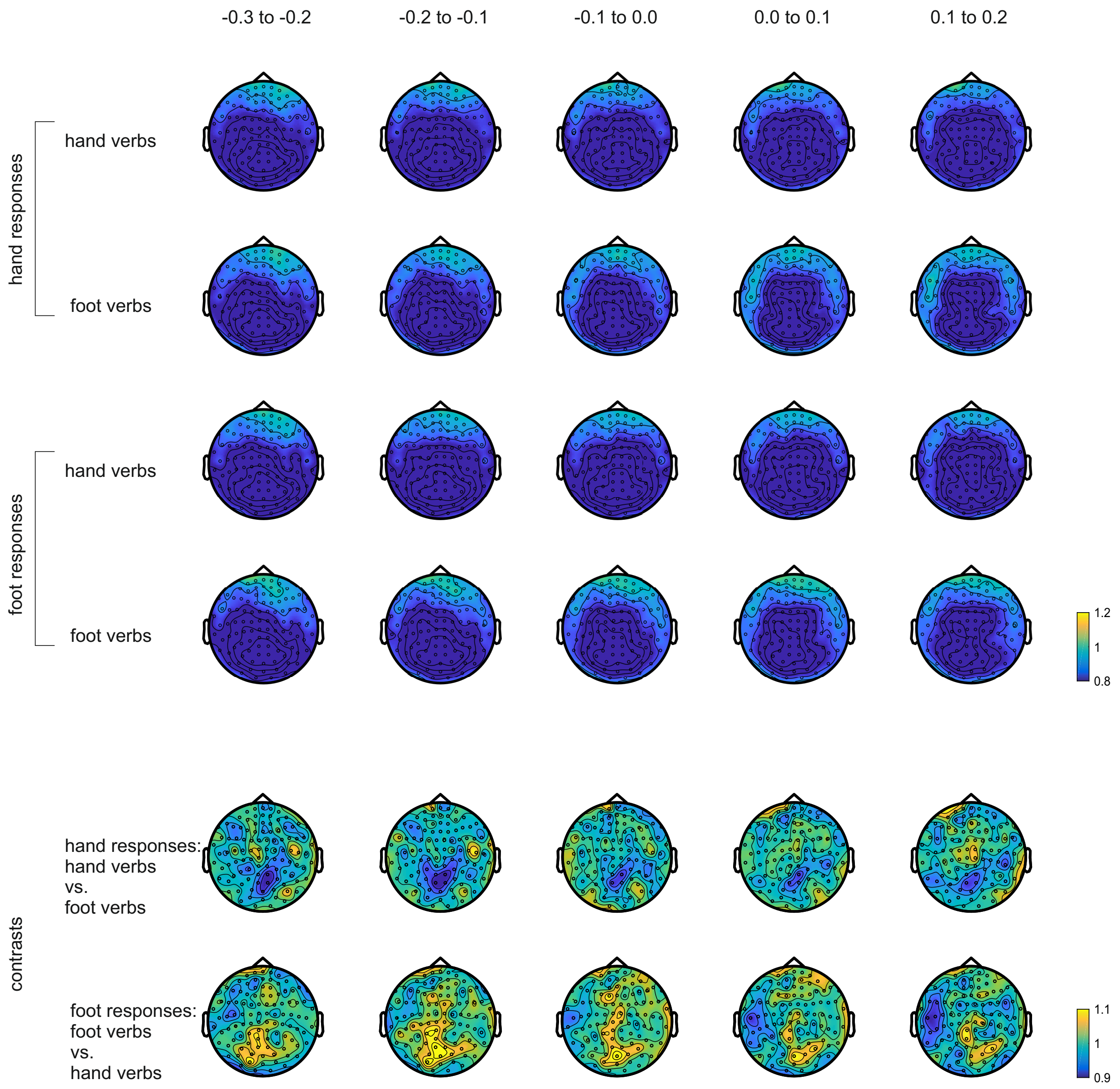

Figure S3: Grandaveraged time-frequency data in all MEG sensors, from -1.3 to 0.2 s in steps of 100 ms, in all four verb-response conditions as well as the contrasts of hand vs. foot verbs within hand responses and foot vs. hand verbs in foot responses. Time is given in seconds before response onset. a-c: alpha band (7-13 Hz), d-f: beta band (20-30 Hz).

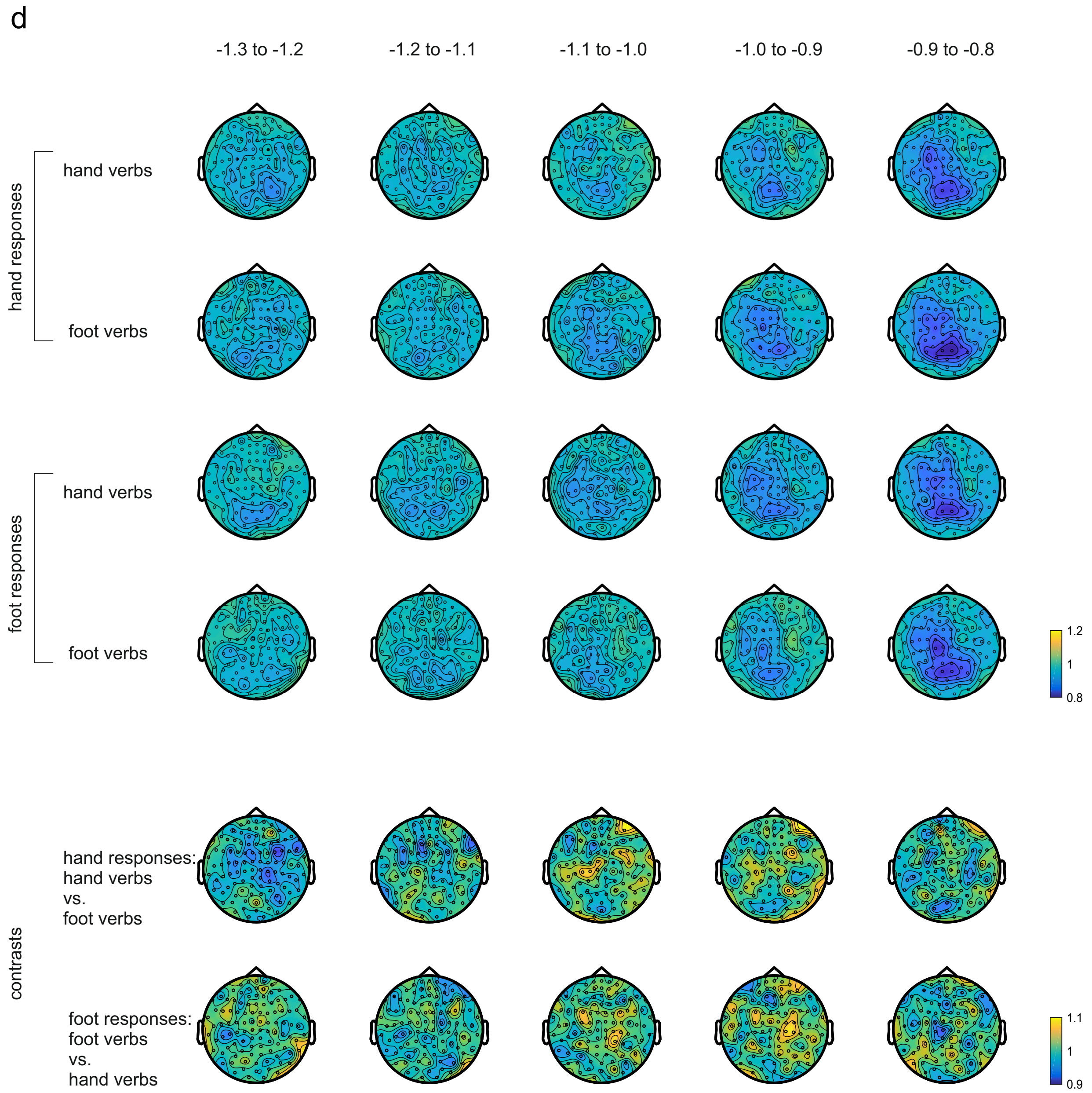

Figure S3: Grandaveraged time-frequency data in all MEG sensors, from -1.3 to 0.2 s in steps of 100 ms, in all four verb-response conditions as well as the contrasts of hand vs. foot verbs within hand responses and foot vs. hand verbs in foot responses. Time is given in seconds before response onset. a-c: alpha band (7-13 Hz), d-f: beta band (20-30 Hz).

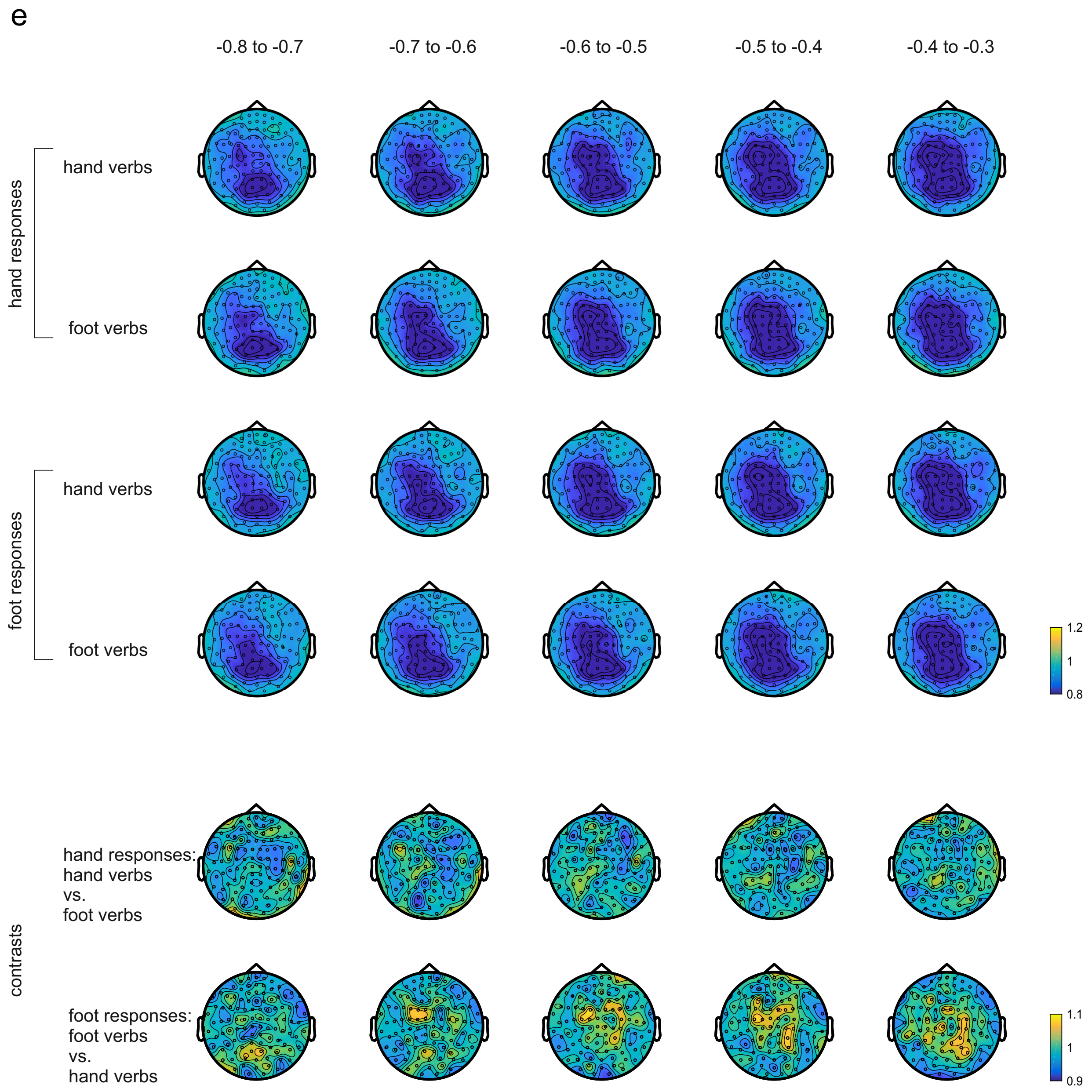

Figure S3: Grandaveraged time-frequency data in all MEG sensors, from -1.3 to 0.2 s in steps of 100 ms, in all four verb-response conditions as well as the contrasts of hand vs. foot verbs within hand responses and foot vs. hand verbs in foot responses. Time is given in seconds before response onset. a-c: alpha band (7-13 Hz), d-f: beta band (20-30 Hz).

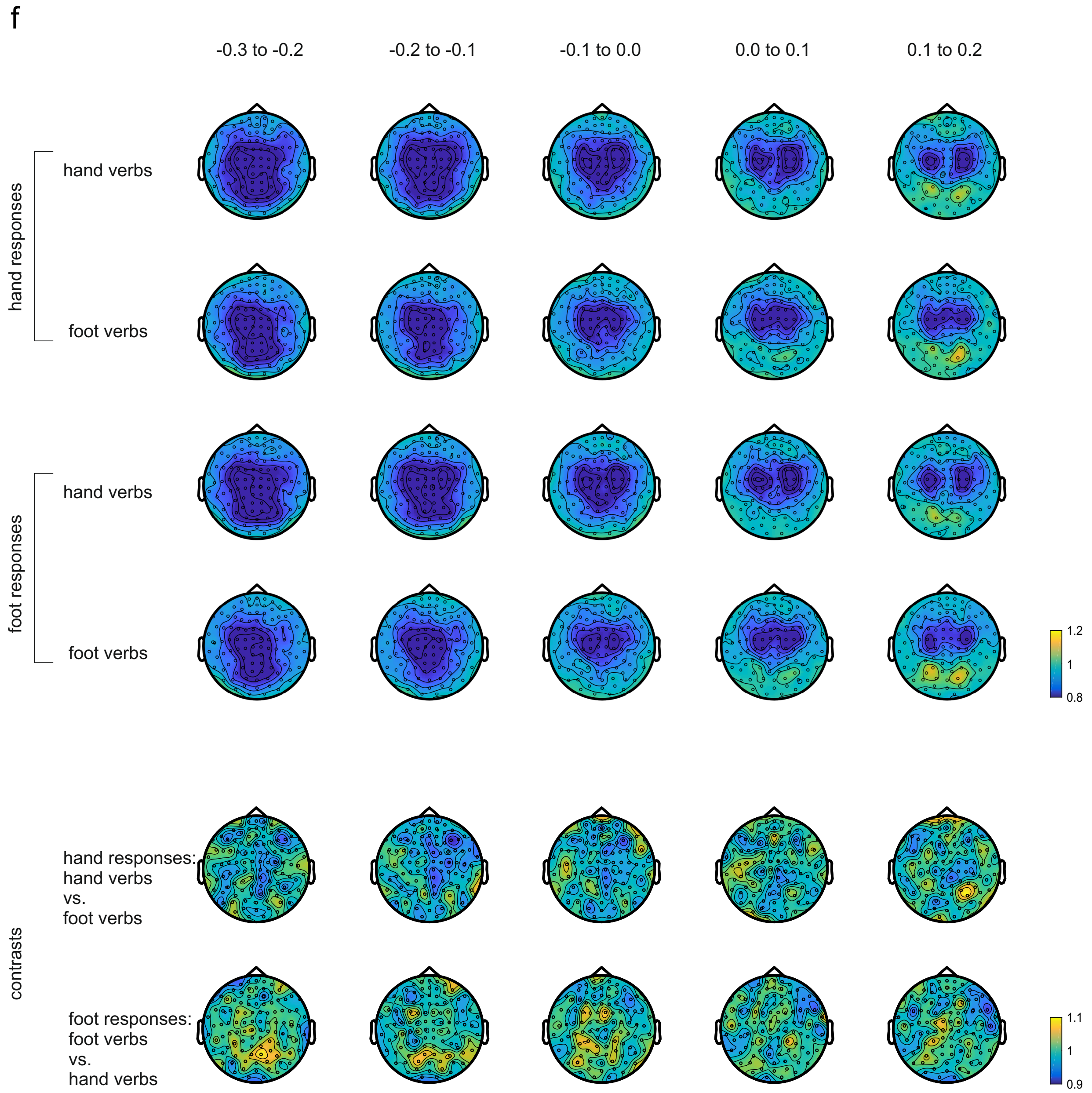

Figure S3: Grandaveraged time-frequency data in all MEG sensors, from -1.3 to 0.2 s in steps of 100 ms, in all four verb-response conditions as well as the contrasts of hand vs. foot verbs within hand responses and foot vs. hand verbs in foot responses. Time is given in seconds before response onset. a-c: alpha band (7-13 Hz), d-f: beta band (20-30 Hz).

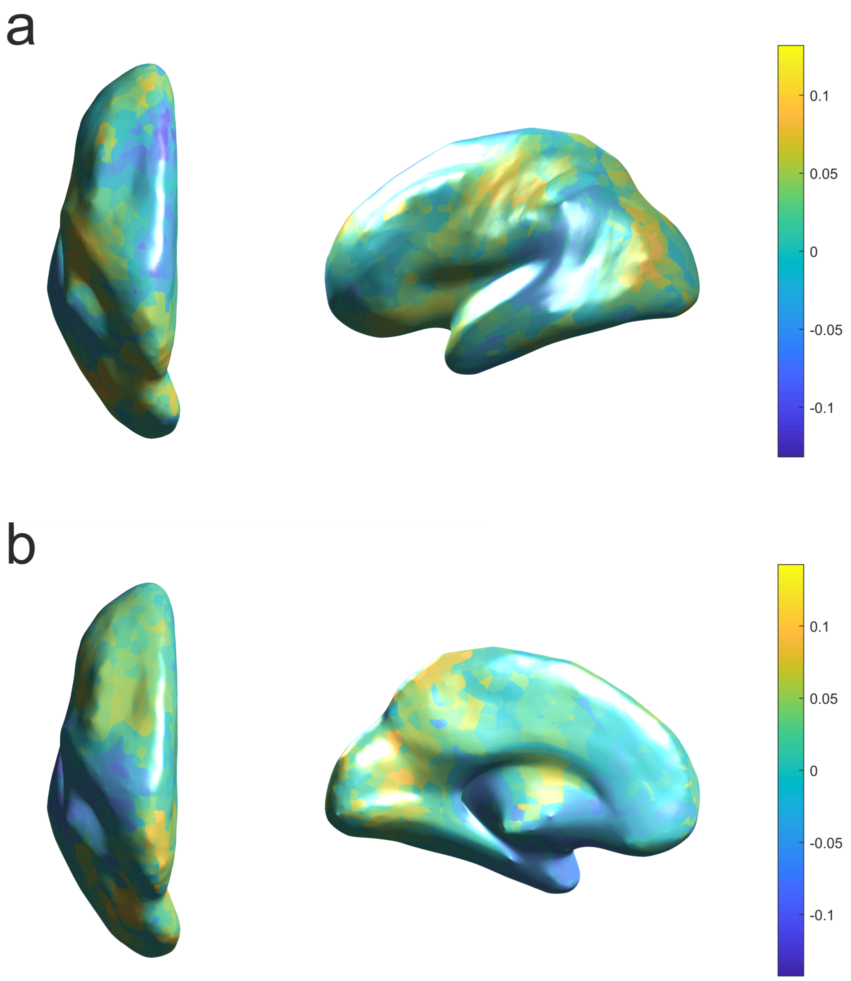

Figure S4: Inflated surface projections of grandaveraged data centered on the time-frequency window of the significant effects. A: within hand responses, hand vs. foot verbs. 7-13 Hz, -1 to -0.5 s before response onset. B: within foot responses, foot vs. hand verbs. 20-30 Hz, -0.3 to 0 s before response onset.

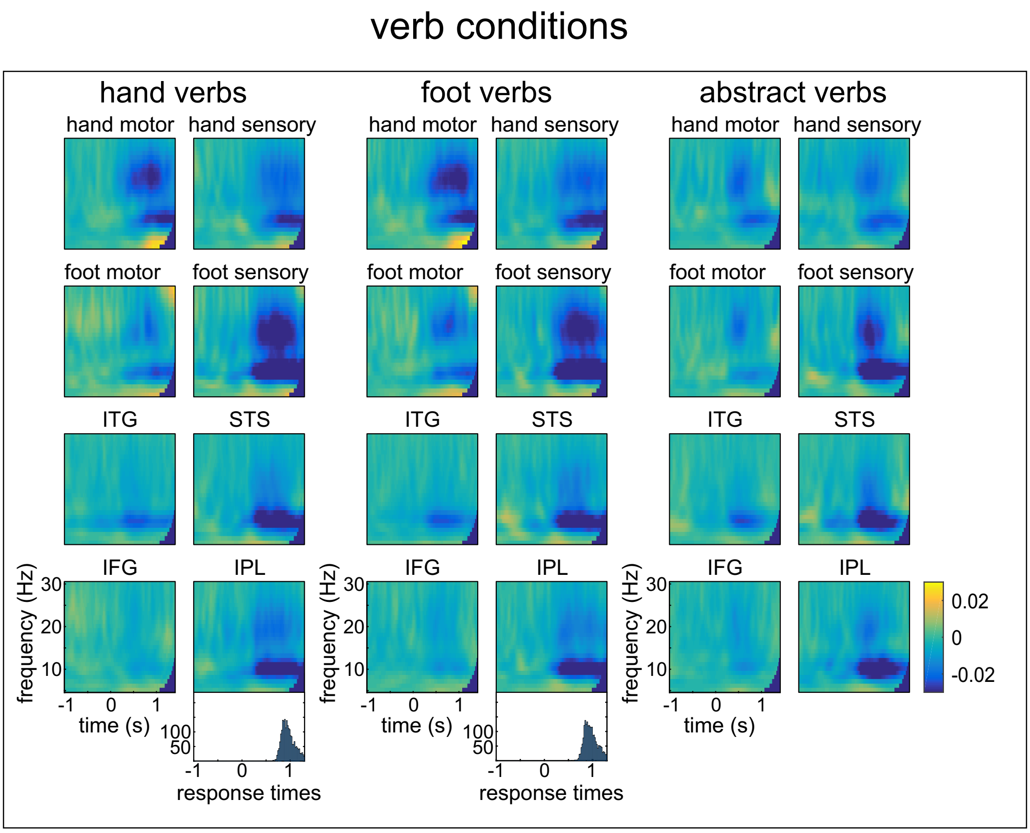

Figure S5: Grandaveraged MEG oscillations in all ROIs in the three verb conditions. Verb onset is 0. A histogram of response times across all trials and all participants is included for hand and foot verb conditions. The response cue had a fixed delay of 400 ms after word onset. Data were baseline-corrected (-1 to -0.5s). ITG = inferior temporal gyrus, STS = superior temporal sulcus, IFG = inferior frontal gyrus, IPL = inferior parietal lobule.

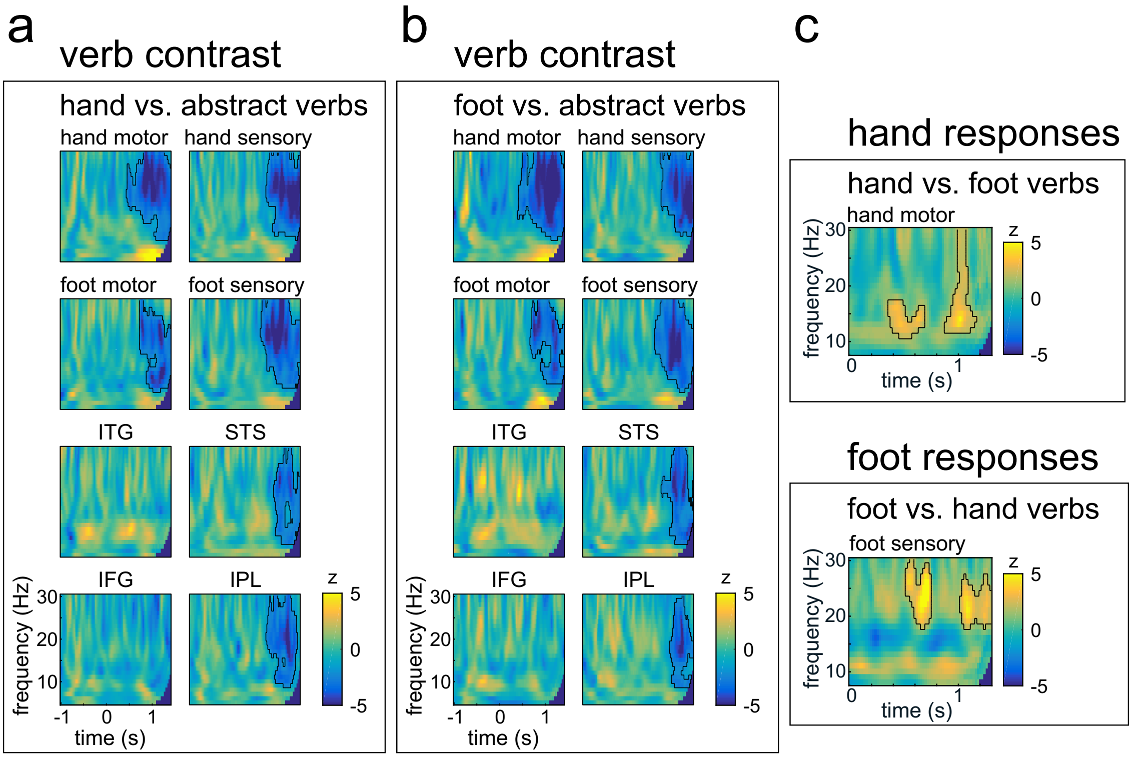

Figure S6: Statistical comparisons of verb-locked MEG data. Significant clusters are outlined. Verb onset is 0. ITG = inferior temporal gyrus, STS = superior temporal sulcus, IFG = inferior frontal gyrus, IPL = inferior parietal lobule.
